# Development of influenza A(H7N9) candidate vaccine viruses with improved hemagglutinin antigen yield in eggs

**DOI:** 10.1111/irv.12322

**Published:** 2015-08-04

**Authors:** Callie Ridenour, Adam Johnson, Emily Winne, Jaber Hossain, Guaniri Mateu-Petit, Amanda Balish, Wanda Santana, Taejoong Kim, Charles Davis, Nancy J Cox, John R Barr, Ruben O Donis, Julie Villanueva, Tracie L Williams, Li-Mei Chen

**Affiliations:** a Influenza Division, National Center for Immunization and Respiratory Diseases, Centers for Disease Control and PreventionAtlanta, GA, USA; b Division of Laboratory Sciences, National Center for Environmental Health, Centers for Disease Control and PreventionAtlanta, GA, USA; c Battelle Memorial InstituteAtlanta, Georgia, USA

**Keywords:** Antigen yield, H7N9, hemagglutinin, influenza, serial passage, vaccine

## Abstract

**Background:**

The emergence of avian influenza A(H7N9) virus in poultry causing zoonotic human infections was reported on March 31, 2013. Development of A(H7N9) candidate vaccine viruses (CVV) for pandemic preparedness purposes was initiated without delay. Candidate vaccine viruses were derived by reverse genetics using the internal genes of A/Puerto/Rico/8/34 (PR8). The resulting A(H7N9) CVVs needed improvement because they had titers and antigen yields that were suboptimal for vaccine manufacturing in eggs, especially in a pandemic situation.

**Methods:**

Two CVVs derived by reverse genetics were serially passaged in embryonated eggs to improve the hemagglutinin (HA) antigen yield. The total viral protein and HA antigen yields of six egg-passaged CVVs were determined by the BCA assay and isotope dilution mass spectrometry (IDMS) analysis, respectively. CVVs were antigenically characterized by hemagglutination inhibition (HI) assays with ferret antisera.

**Results:**

Improvement of total viral protein yield was observed for the six egg-passaged CVVs; HA quantification by IDMS indicated approximately a twofold increase in yield of several egg-passaged viruses as compared to that of the parental CVV. Several different amino acid substitutions were identified in the HA of all viruses after serial passage. However, HI tests indicated that the antigenic properties of two CVVs remained unchanged.

**Conclusions:**

If influenza A(H7N9) viruses were to acquire sustained human-to-human transmissibility, the improved HA yield of the egg-passaged CVVs generated in this study could expedite vaccine manufacturing for pandemic mitigation.

## Introduction

The first human infections with avian influenza A(H7N9) viruses were reported on March 31, 2013. To date, there have been a total of 608 human infections with a 34% fatality rate. The A(H7N9) viruses emerged and spread in birds after reassortment among Eurasia lineage avian influenza viruses from aquatic birds and viruses circulating in domestic poultry, with the former contributing the HA and NA genes and the latter providing the remaining six genes.^1^ The surface and internal genes of A(H7N9) viruses possess several sequence characteristics correlated to increased potential to infect humans and virulence in mammalian hosts. These include mutations in HA that increase human-like receptor specificity as well as mutations in PB2 facilitating replication in the mammalian airway. If A(H7N9) viruses were to acquire sustained human-to-human transmissibility and cause a pandemic, vaccination would be the most important intervention to mitigate its impact on public health.

Pandemic influenza vaccine manufacturing relies on the established seasonal influenza vaccine capacity. Over 90% of vaccine production capacity for the United States consists of inactivated virus propagated in eggs. Wild-type influenza viruses produce low amounts of viral antigen in eggs. Therefore, vaccines are produced using reassortant viruses with the HA and NA genes encoding the antigens eliciting protective immunity to the circulating strains and the internal genes from a laboratory adapted virus imparting high growth in eggs.^2^ The internal genes of A/Puerto Rico/8/1934 (PR8) virus were shown to improve antigen yield and impart an attenuated phenotype to reassortant candidate vaccine viruses (CVV) for vaccine production. Despite the contribution from PR8 genes, some CVVs may not yield the expected amounts of antigen to efficiently produce vaccine and meet pandemic vaccination campaign timetables, which are based on average productivity of seasonal influenza vaccines.^3^–^6^ In this report, we describe the development and characterization of reassortant A(H7N9) viruses and the constellation of amino acid substitutions associated with improved antigen yield resulting from serial propagation in the allantoic sac of embryonated eggs.

## Materials and methods

### Generation and sequence analysis of reassortant viruses by reverse genetics

The derivation and propagation of the reassortant virus were performed in accordance with WHO guidance for development of vaccine reference viruses.^7^ Reassortant viruses were generated from plasmids by reverse genetics methods.^8^ The HA and NA genes of the A/Shanghai/2/2013 virus were amplified by PCR (polymerase chain reaction) from synthetic DNA amplicons (kindly provided by Novartis Vaccines and Diagnostics), including a single nucleotide polymorphism (SNP) in the H7 and N9 untranslated regions (UTR); sequences available upon request. The genes were cloned into a reverse genetics vector flanked by human polymerase I promoter and mouse RNA polymerase I terminator elements.^9^ Reverse genetics plasmids encoding the HA and NA surface genes as well as plasmids containing the six internal genes from PR8 were transfected into qualified Vero cells from a cell bank system using Lipofectamine 2000 (Life Tech, Grand Island, NY, USA).^10^ Viruses from transfected Vero cells were propagated in the allantoic sac of 10- to 12-day-old specific pathogen-free embryonated hen eggs (Charles Rivers Laboratories, North Franklin, CT, USA) using limiting dilution and incubated at 35–37°C for 48 hours to prepare virus stocks. To establish serial propagation of virus, allantoic fluid testing positive for hemagglutination activity (3–5 eggs pooled) was used to directly inoculate eggs of each subsequent passage using the methods described above.

Total RNA was extracted from allantoic fluid using the QiaAmp Viral RNA minikit (Qiagen; Valencia, CA), reverse-transcribed to cDNA, and amplified using a one-step reaction system (One-step RT-PCR kit; Qiagen, Valencia, CA, USA) using sequence-specific primers. Sequence analysis of the resulting DNA amplicons served as templates for automated sequencing on an Applied Biosystems 3130 genetic analyzer, using cycle sequencing dye terminator chemistry (BigDye terminator v3·1 cycle sequencing kit; Life Tech).

### Concentration of reassortant viruses for protein yield analysis

Reassortant viruses were grown in 10 to 11-day-old embryonated hen eggs at 35°C for 60–64 hours. Allantoic fluid was harvested from the chilled eggs and clarified at 5400 × ***g***, 10 minutes at 4°C (Sorvall SLA-1500 rotor). The supernatant was collected and inactivated with ß-propiolactone (BPL) for approximately 24 hours at 4°C before further purification. The inactivated virus was clarified twice more at 15,000 × ***g***, 5 minutes at 4°C (Sorvall SLA-1500 rotor). Virus was pelleted by centrifugation at 39,000 × ***g***, 3 hours at 4°C (Sorvall A621 rotor). Virus pellets were resuspended overnight in PBS and loaded onto a 30%/55% (w/w) density sucrose gradient. The gradient was centrifuged at 90,000 × ***g*** for 14 hours at 4°C (Sorvall AH629 rotor). The virus fractions were harvested and sedimented at 131,000 × ***g*** (Sorvall AH629 rotor) for 2·5 h. At least two independent virus concentrates were generated for each virus.

### Quantification of total viral protein in virus samples by BCA

A microplate BCA assay kit (Pierce, Rockford, IL, USA) was used to measure the total protein content of purified viruses. NP40 was added to the samples to a final concentration of 0·25%, and the BCA assay was performed according to manufacturer’s instructions. Bovine serum albumin (BSA) provided in the kit was used as the standard curve, and absorbance was read at 560 nm.

### Quantification of HA, NP, and M1 by IDMS

Viral samples for analysis by isotope dilution mass spectrometry (IDMS) were digested with trypsin. The resulting peptide fragments were separated by liquid chromatography and quantified by IDMS.^11^ Four peptides were used for the quantification of HA of the H7 subtype: VNTLTER_34-40_, FVNEEALR_119-126_, IQIDPVK_510-516_, and STQSAIDQITGK_379-390._^12^ Two peptides, LIQNSLTIER and GVFESLDEK, were used as the standards for NP quantification, and peptide EITFHGAK was the standard for M1 protein measurement.

### Immunization of ferret and preparation of antisera

All animal study protocols were reviewed and approved by CDC’s Institutional Animal Care and Use Committee (IACUC) and in compliance of animal welfare and biosafety requirements. Antisera to reassortant viruses were produced in ferrets pre-screened for absence of antibodies to seasonal influenza viruses. Two ferrets were inoculated with virus diluted 1:10 in 0·85% physiological saline (500 μl per nostril). A blood sample collected on day 13 or 14 post-inoculation (PI) was tested in a hemagglutination inhibition (HI) assay to determine whether boosting was required. On day 15 PI, ferrets were boosted intradermally with concentrated virus containing adjuvant (Titermax; Sigma, St. Louis, MO, USA) if the pre-boost titers were <80. Blood was collected on day 28 PI. Both treatment and testing of serum were performed according to Klimov *et al*.^13^

## Results

### Generation of H7N9 PR8 reassortant viruses by reverse genetics

To produce A(H7N9) CVV with high growth properties, we derived reassortant viruses with the HA/NA surface genes from A/Shanghai/2/2013 (H7N9) virus and internal genes from PR8 virus. To expedite candidate vaccine virus development while biological materials were being shipped internationally, the HA and NA coding sequences were synthesized based on data provided by the Chinese National Influenza Center. Viruses were recovered from transfected Vero cells by inoculation and propagation in embryonated eggs. The complete genomic sequences from the second egg passage (V1E2) of two reassortant viruses, IDCDC-RG32A and IDCDC-RG32B (with SNP in HA/NA UTR), were analyzed. Analysis indicated absolute identity with the influenza gene sequences from the reverse genetics plasmids, including the UTR. The HA titers of both the IDCDC-RG32A and IDCDC-RG32B V1E2 stocks were 256 using turkey red blood cells, with a median egg infectivity (EID_50_) of 10^9·4^/ml for both virus stocks.

### Total protein and HA antigen yield analysis of IDCDC-RG32A and IDCDC-RG32B

**Figure 1:**
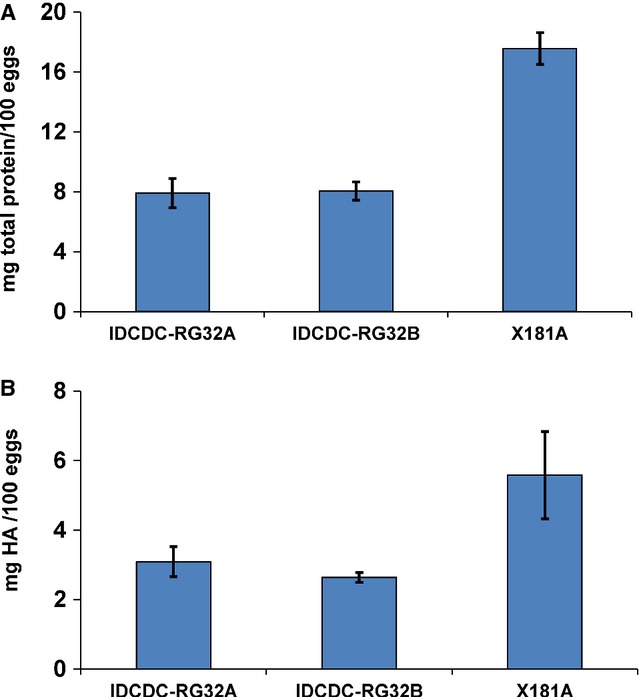
Quantification of viral protein as compared to the high growth A(H1N1)pdm09 reassortant X-181A. (A) Quantification of total viral protein, shown as mg total viral protein/100 eggs. (B) Quantification of HA antigen, shown as mg HA/100 eggs. Values shown are the average of at least two independent experiments with errors bars denoting standard deviation.

The HA content in virus purified from eggs is a critical attribute of any candidate vaccine virus to be used in manufacturing vaccine for an urgent pandemic response. Although the infectivity and HA titers of a virus in the allantoic fluid can give an indication of the total virus concentration, these parameters are not reliable indicators of HA content in purified viruses. To better estimate the HA content of the A(H7N9) PR8 reassortants, we analyzed the total viral protein and HA antigen yield of inactivated virions purified from allantoic fluid by differential and equilibrium ultracentrifugation in sucrose gradients. In this study, both IDCDC-RG32A and IDCDC-RG32B viruses yielded approximately 8 mg virus protein/100 eggs (based on an average of 9 mL of allantoic fluid collected per egg). This yield is lower than the minimal requirement for vaccine manufacturing, representing only 45% of the total protein yield obtained under identical conditions from an A(H1N1)pdm09 reassortant virus X-181A, with an average yield for a seasonal influenza CVV (Figure1A). To quantify the HA antigen yield, we analyzed purified virus samples by isotope dilution mass spectrometry (IDMS).^11^ Four peptides of H7 HA (two located on HA1 and two on HA2) were used as specific target peptides that are stoichiometric representatives of the HA protein, as previously described.^12^ Multiple target peptides were used to ensure that enzymatic digestion of the protein was complete and reproducible as well as to verify accuracy of the measurement.^11^,^12^ The IDMS data indicated no significant HA protein yield differences between purified IDCDC-RG32A and IDCDC-RG32B viruses, with an average of 2·9 ± 0·2 mg HA/100 eggs. In contrast, the antigen yield of X-181A averaged 5·6 ± 1·2 mg HA/100 eggs, approximately a twofold greater yield than the H7N9 PR8 reassortants (Figure1B). Therefore, the antigen yields of both IDCDC-RG32A and IDCDC-RG32B viruses were considered suboptimal for vaccine manufacturing in eggs.

### Serial Passage of A(H7N9) PR8 reassortants in eggs

**Figure 2:**
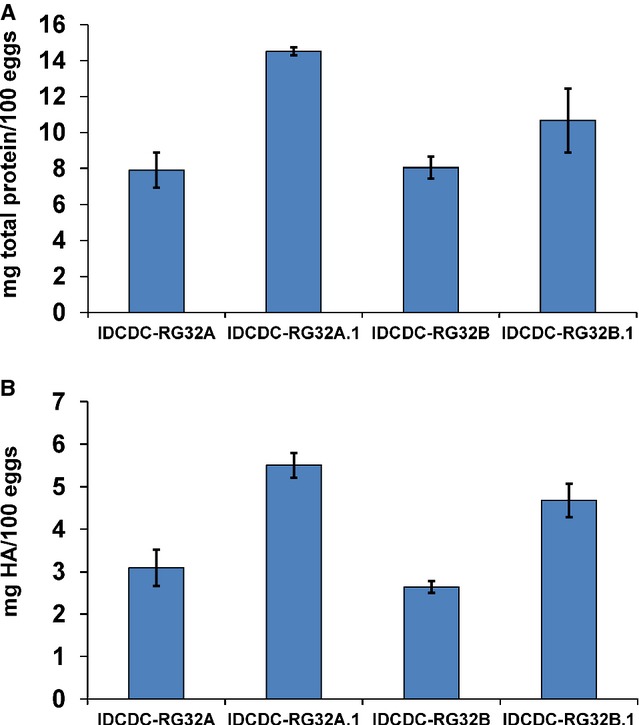
Yield analysis of egg-passaged reassortant viruses as compared to parental virus yield (A) Quantification of total viral protein, shown as mg total viral protein/100 eggs. (B) Quantification of HA antigen, shown as mg HA/100 eggs. Values shown are the average of at least two independent experiments with errors bars denoting standard deviation.

Candidate vaccine viruses used to manufacture seasonal influenza vaccines undergo approximately four to five egg passages in the course of classical reassortment with PR8, with several additional passages in eggs to achieve higher titers and establish the master and working seed stocks for manufacturing.^14^ Dozens of CVVs prepared since the late 1970′s validate this approach for development of high yield viruses.^15^ In contrast to seasonal CVVs prepared by classical reassortment in eggs, pandemic CVVs derived by reverse genetics are generally passaged only twice in eggs prior to establishment of master and working seed stocks.^16^ In this study, we used serial passage in eggs as an approach to improve the antigen yields of the reverse genetics derived A(H7N9) PR8 reassortant viruses. Genetic analysis of the HA and NA genes from IDCDC-RG32B V1E13 (initial growth in Vero cells followed by 13 passages in eggs) stocks revealed a mixture of Gly/Glu at codon 196 of the mature HA protein (Gly as the wild-type codon, Glu as the mutant). Although no mutations in HA or NA genes were detected in the IDCDC-RG32A V1E13 virus stock, a mixture of Asn/Asp was detected at HA codon 149 in the V1E16 passage stock. Residue 149 (158 in H3 numbering) is located at the top of the globular head and has previously been shown to undergo positive selection in subtype H3 viruses when adapted to eggs.^17^ Residue 196 is at the monomer interface and has been shown to modulate receptor binding in the adjacent monomer.^18^,^19^ In order to evaluate the impact of these HA mutations on antigen yield, virus stocks with homogeneous sequences were prepared by limiting dilution and designated IDCDC-RG32A.1 (with 149Asp in HA) and IDCDC-RG32B.1 (with 196Glu in HA). The total viral protein yield of IDCDC-RG32A.1 and IDCDC-RG32B.1 after sucrose density gradient separation was 14·5 ± 0·2 mg virus protein/100 eggs and 10·7 ± 1·8 mg virus protein/100 eggs, respectively. The HA antigen yield of IDCDC-RG32A.1 and IDCDC-RG32B.1 was 5·5 ± 0·3 mg HA/100 eggs and 4·7 ± 0·4 mg HA/100 eggs, respectively, approximately a 78% and 81% increase in HA yield compared to their respective parental virus (Figure2).

To investigate whether further egg passage of the IDCDC-RG32A.1 or IDCDC-RG32B.1 stocks could provide an additional boost in antigen yields, we performed multiple independent egg passage experiments using IDCDC-RG32A.1 and IDCDC-RG32B.1 viruses, creating a second generation of egg-passaged reassortant viruses. After an additional ten to thirteen passages, the HA titer of each passaged virus increased from 512 to 1024 (Table1). Increases in median egg infectivity (EID_50_/ml) of both the IDCDC-RG32A and IDCDC-RG32B viruses were also observed (Table1). Additional mutations were identified in the HA genes of these passaged viruses (Table1). The second-generation IDCDC-RG32A.2 and IDCDC-RG32A.3 viruses that were derived from IDCDC-RG32A.1 featured the HA substitutions Gly196Glu (RG32A.2) and Leu217Gln (RG32A.3) in addition to the original Asn149Asp change. In addition to the original G196E substitution, passaged viruses derived from IDCDC-RG32B.1 also acquired additional substitutions at or near the receptor binding site or antigenic sites, including 1-2 amino acid substitutions at residues 89, 96, 130, 189, and/or 217.

**Table 1:** Properties of A(H7N9) PR8 CVV after serial passage in eggs

Virus	Sequence Changes in HA*							EID_50/_ml**	HA titer
IDCDC-RG32A.1	N149D	–	–	–	–	–	–	10^9·5^	1024
IDCDC-RG32A.2	N149D	G196E	–	–	–	–	–	10^9·7^	1024
IDCDC-RG32A.3	N149D	–	L217Q					10^9·6^	1024
IDCDC-RG32B.1	–	G196E	–	–	–	–	–	10^9·9^	1024
IDCDC-RG32B.2	–	G196E	–	G189E	E96K	–	–	10^10^	1024
IDCDC-RG32B.3	–	G196E	–	–	–	R130M	–	10^9·7^	512
IDCDC-RG32B.4	–	G196E	L217Q	–	–	–	P89S	10^9·2^	1024
IDCDC-RG32B.5	–	G196E	–	G189E	–	–	–	10^9·5^	1024

Underlined text indicates a virus stock from which subsequent viruses were derived (rows below).

* Mature H7 HA numbering system was used.

** EID_50_ was calculated according to Reed and Muench method.

**Figure 3:**
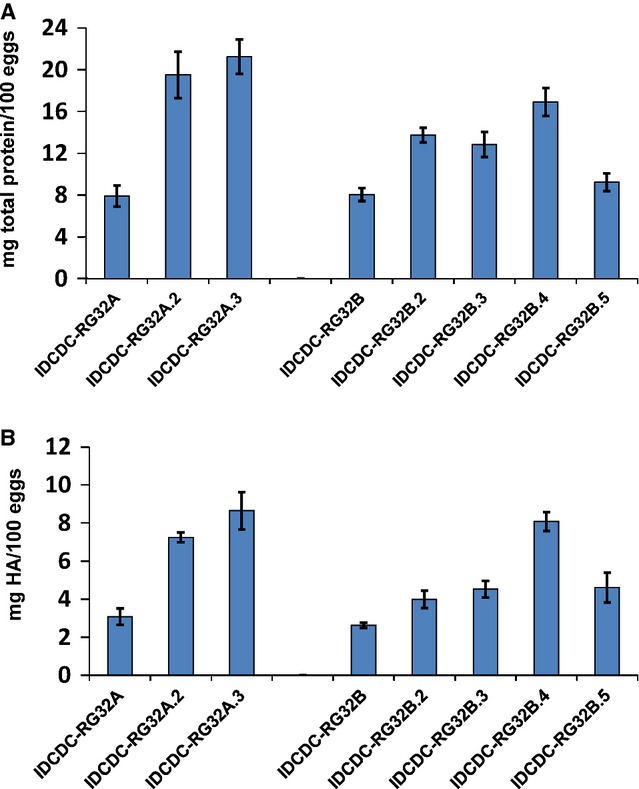
Yield analysis of second-generation reassortant viruses derived from parental CVV. (A) Quantification of total viral protein, shown as mg total viral protein/100 eggs (B) Quantification of HA antigen, shown as mg HA/100 eggs. Values shown are the average of at least two independent experiments with errors bars denoting standard deviation.

The total viral protein yield of sucrose gradient-purified samples from the second-generation viruses ranged from 9 to 21 mg protein/100 eggs (Figure3A), with HA yields of 4–9 mg/100 eggs quantified through IDMS (Figure3B). Notably, three of the six viruses, IDCDC-RG32A.2, IDCDC-RG32A.3, and IDCDC-RG32B.4, achieved total viral protein and HA antigen yields comparable to those of the seasonal high growth reassortant, X-181A (H1N1)pdm09. The HA/NP and HA/M1 molar ratios from all egg-passaged viruses remained similar to the HA/NP and HA/M1 ratios of their respective parental virus (Table2).

**Table 2:** Viral protein molar ratios and HA content of concentrated virions

Virus	HA/NP ratio*	HA/M1 ratio*	HA(%)**
IDCDC-RG32A	2·26	0·79	39·4
IDCDC-RG32A.1	1·97	0·62	37·9
IDCDC-RG32A.2	1·88	0·72	37·7
IDCDC-RG32A.3	1·87	0·71	39·6
IDCDC-RG32B	1·91	0·78	32·8
IDCDC-RG32B.1	2·10	0·86	44·9
IDCDC-RG32B.2	1·92	0·79	28·9
IDCDC-RG32B.3	2·17	0·89	35·4
IDCDC-RG32B.4	1·85	0·80	48·0
IDCDC-RG32B.5	2·18	0·89	49·6

Underlined text as in Table1.

Values shown are the average of at least two independent experiments.

* Viral protein concentrations were determined by IDMS as described in Methods.

** Calculated as follows: HA protein by IDMS/total protein by BCA assay.

### Antigenic analysis of egg-passaged reassortant viruses

If the amino acid changes in the HA of serially passaged, egg-adapted CVVs were to alter the antigenic properties of the virus, the CVVs would no longer be considered acceptable for vaccine manufacturing as the product would be rejected by national vaccine regulatory authorities.^20^ The antigenic characteristics of the egg-passaged CVVs were analyzed by HI assays using a panel of ferret antisera raised against parental viruses as well as the egg-adapted viruses. As A/Anhui/1/2013 and A/Shanghai/2/2013 viruses encode identical amino acid sequences of their HA gene, the A/Anhui/1/2013 virus was used as the wild-type reference virus in our HI analyses. Antisera to A/Anhui/1/2013 inhibited all of the egg-passaged CVV with titers of less than or equal to twofold difference from that with the homologous virus (data not shown). Two of the CVV with the highest total viral proteins yields, IDCDC-RG32A.2 and IDCDC-RG32A.3, along with the parental IDCDC-RG32A were further selected to immunize ferrets to generate antisera. The titer of antiserum to IDCDC-RG32A.2 tested against A/Anhui/1/2013 virus (surrogate of wt parent) was fourfold lower than that of homologous virus antigen (Table3), indicative of significant antigenic differences with the parental virus. In contrast, the titers of ferret antisera to both IDCDC-RG32A and IDCDC-RG32A.3 viruses tested against A/Anhui/1/2013 virus were equivalent (less than or equal to twofold difference) to those with their homologous antigens. These findings indicate that IDCDC-RG32A and IDCDC-RG32A.3 retain the antigenic characteristics of the parental A/Shanghai/2/2013, represented by A/Anhui/1/2013 virus.

**Table 3:** Antigenic analysis of candidate vaccine viruses by hemagglutination inhibition

Virus	Ferret antiserum			
	A/Anhui/1/13*	IDCDC-RG32A*,**	IDCDC-RG32A.2*	IDCDC-RG32A.3*
A/Anhui/1/2013	**160**	320	40	80
IDCDC-RG32A	320	**640**	40	160
IDCDC-RG32A.2	320	640	**160**	640
IDCDC-RG32A.3	80	160	80	**160**

Boldface/underlined values denote titers of ferret sera with homologous antigens.

* Ferret antisera was collected after antigen boost by intradermal injection.

** Ferret was boosted with adjuvanted antigen.

## Discussion

Antigenic characterization of the A(H7N9) virus with post-infection ferret sera revealed significant antigenic differences from the Eurasian and North American subtype H7 CVV developed previously.^21^ CVVs developed by reassortment using reverse genetics based on the WHO-recommended A/Anhui/1/2013-like (H7N9) virus provide a more suitable alternative for vaccine production. The internal genes from the PR8 virus were used to enhance their growth in eggs and to attenuate virulence.^16^ Surprisingly, the HA titers of the IDCDC-RG32A and IDCDC-RG32B A(H7N9) PR8 reassortant CVVs were much lower than that of another Eurasia H7 candidate vaccine virus with the HA gene from the A/mallard/Netherlands/12/2000 virus.^22^ The low HA titers of these A(H7N9) reassortant CVVs were consistent with their low total viral protein yields as well as their low estimated HA yields of approximately 3·0 mg HA/100 eggs, falling well below the average manufacturing benchmark of approximately 4–5 mg HA/100 eggs.^23^

A previous report has indicated that sequential passage of CVVs in eggs resulted in substantially higher antigen yields.^6^ Improved growth of these influenza A(H1N1)pdm09 CVVs in eggs was associated with amino acid substitutions in the HA glycoprotein. Vaccine manufacturers rely on egg-adapted CVVs for production because they yield higher virus titers and increased amounts of antigen. The three egg-adapted viruses derived from IDCDC-RG32A and the five egg-adapted viruses derived from IDCDC-RG32B revealed improved growth in eggs with a set of HA codon changes that could potentially modulate virus–cell interactions. The HA titers of the 6-second-generation passaged viruses were 1024, and HA antigen production from five of the six viruses ranged from 5 to 9 mg HA/100 eggs. Despite individual variation, this HA yield is estimated to produce approximately 3–6 monovalent doses (15 μg/dose) of influenza vaccine per egg, similar to the yield required for seasonal influenza vaccine production.^23^

The three viruses with the highest HA antigen yield, IDCDC-RG32A.2, IDCDC-RG32A.3, and IDCDC-RG32B.4, possess HA substitutions at residues 149, 196, 217, and/or 89. Although formal demonstration of their individual and combinatorial roles in virus–host interactions would require additional experiments, previous studies suggest such a possibility. Gly196 is buried at the monomer interface and is structurally equivalent to Ser205 at antigenic site D of H3 HA.^19^ Despite not being part of the receptor binding pocket structure, its proximity to the receptor pocket entrance on the neighboring HA monomer likely influences glycan interactions.^19^,^24^ A recent study demonstrated that a Gly196Glu substitution in Eurasian H7N1 viruses (G205E in H3 numbering) influenced sialoglycan binding specificity.^18^ Asn149 is located at the top of the HA globular head and is structurally equivalent to Gly158 at antigenic site B of the H3 HA. Earlier studies identified a change at this position in human A(H3N2) viruses adapted to eggs^17^ and demonstrated its impact on interactions with sialylglycolipid ligands.^25^ Besides their roles in HA-glycan receptor interactions, the Asn149Asp and Gly196Glu substitutions increase the local negative charge of HA altering the electromagnetic field around the top of the HA head, thereby modulating electrostatic interactions of virions with host cell surfaces, that is, epithelial cells in the allantoic sac, and multicycle virus replication.^26^,^27^ Leu217 is located on the side wall of the receptor binding pocket and is structurally equivalent to Leu226 in human seasonal H3N2 viruses.^28^,^29^ The Gln226Leu substitution was essential to enable avian influenza viruses to infect and transmit among humans in the 1957 A(H2N2) and 1968 A(H3N2) pandemics. All subtype H7 viruses isolated from birds before 2013 have Gln217. In contrast, many chicken isolates and the majority of the human H7N9 isolates detected since 2013 have Leu217, including the A/Shanghai/2/2013 virus. Influenza A Leu217 viruses bind to a reduced subset of α2,3-linked sialylglycans, as compared to their Gln217 counterparts.^28^,^29^ Egg passage of both IDCDC-RG32A and IDCDC-RG32B viruses resulted in Leu217Gln codon substitutions. This reversion to the avian-like sequence suggests that improved binding to α2,3-linked sialylglycans may have enhanced growth in eggs. The close proximity of antigenic sites to the receptor binding pocket of HA increases the probability that mutations at or near the pocket leading to changes in receptor binding may have the unintended consequence of altering antigenicity.^30^ Chen *et al*. reported that Asn149Asp (antigenic site A) altered the antigenic properties of an A(H7N9) virus.^31^ The Asn149Asp and Gly196Glu mutations (antigenic sites B and D) of RG32A.2 selected by egg passage in this study resulted in antigenic changes detectable by reciprocal HI testing. On the other hand, ferret antisera to IDCDC-RG32A.3 virus indicated that this virus retained the antigenic characteristics of the WHO-recommended wild-type virus, suggesting that the Asn149Asp mutation does not alter the antigenic characteristics if paired with a Leu217Gln substitution, emphasizing the importance of structural context to assess functional significance.

No amino acid changes were detected in the internal genes of the first-generation passaged viruses, and 5 of the 6 second-generation viruses had only a single coding change (RG32A.3: PA-K113Q, RG32B.2: NS1-A122S, RG32B.3: PB2-I540D, RG32B.4: NP-A286S, RG32A.5: PB1-R505W). This is consistent with a previous study that reported a low frequency of changes in these genes as a result of their extensive adaptation to eggs.^32^ Some amino acid substitutions were identified in the NA genes of the A(H7N9) PR8 reassortant egg-passaged viruses. Compensatory changes in NA have often been detected when HA undergoes host selection.^33^–^35^ In this case, NA mutations could be necessary for optimized functional balance between HA and NA during growth in eggs. The role of these NA changes in antigen yield improvement will be addressed in future studies.

Several A(H7N9) reassortant viruses with improved vaccine yield have been prepared and could be considered for vaccine production for pandemic preparedness if they meet the required HA yield in eggs as well as retain antigenic similarity to the circulating poultry viruses. One of these viruses, IDCDC-RG32A.3, has an HA yield comparable to that required for efficient production of seasonal influenza vaccines and has retained the antigenic characteristics of the wild-type virus. If H7N9 viruses were to acquire sustained human-to-human transmissibility, the improved HA yield of this A(H7N9) candidate vaccine virus could expedite vaccine manufacturing for pandemic mitigation.

## References

[b1] Liu D, Shi W, Shi Y (2013). Origin and diversity of novel avian influenza A H7N9 viruses causing human infection: phylogenetic, structural, and coalescent analyses. Lancet.

[b2] Kilbourne ED, Schulman JL, Schild GC, Schloer G, Swanson J, Bucher D (1971). Related studies of a recombinant influenza-virus vaccine. I. Derivation and characterization of virus and vaccine. J Infect Dis.

[b3] Harvey R, Wheeler JX, Wallis CL, Robertson JS, Engelhardt OG (2008). Quantitation of haemagglutinin in H5N1 influenza viruses reveals low haemagglutinin content of vaccine virus NIBRG-14 (H5N1). Vaccine.

[b4] Abt M, de Jonge J, Laue M, Wolff T (2011). Improvement of H5N1 influenza vaccine viruses: influence of internal gene segments of avian and human origin on production and hemagglutinin content. Vaccine.

[b5] Jing X, Phy K, Li X, Ye Z (2012). Increased hemagglutinin content in a reassortant 2009 pandemic H1N1 influenza virus with chimeric neuraminidase containing donor A/Puerto Rico/8/34 virus transmembrane and stalk domains. Vaccine.

[b6] Robertson JS, Nicolson C, Harvey R (2011). The development of vaccine viruses against pandemic A(H1N1) influenza. Vaccine.

[b7] WHO (2005). http://www.who.int/csr/resources/publications/influenza/WHO_CDS_CSR_GIP_2005_6.pdf.

[b8] Hoffmann E, Krauss S, Perez D, Webby R, Webster RG (2002). Eight-plasmid system for rapid generation of influenza virus vaccines. Vaccine.

[b9] Dong J, Matsuoka Y, Maines TR (2009). Development of a new candidate H5N1 avian influenza virus for pre-pandemic vaccine production. Influenza Other Respir Viruses.

[b10] CBER/FDA, US Department of Health and Human Services (2010). Guidance for Industry on Characterization and Qualification of Cell Substrates and Other Biological Materials Used in the Production of Viral Vaccines for Infectious Disease Indications.

[b11] Williams TL, Luna L, Guo Z (2008). Quantification of influenza virus hemagglutinins in complex mixtures using isotope dilution tandem mass spectrometry. Vaccine.

[b12] Santana WI, Williams TL, Winne EK, Pirkle JL, Barr JR (2014). Quantification of viral proteins of the avian H7 subtype of influenza virus-an isotope dilution mass spectrometry method applicable for producing more rapid vaccines in the case of an influenza pandemic. Anal Chem.

[b13] Klimov A, Balish A, Veguilla V (2012). Influenza virus titration, antigenic characterization, and serological methods for antibody detection. Methods Mol Biol.

[b14] Stohr K, Bucher D, Colgate T, Wood J (2012). Influenza virus surveillance, vaccine strain selection, and manufacture. Methods Mol Biol.

[b15] Fulvini AA, Ramanunninair M, Le J (2011). Gene constellation of influenza A virus reassortants with high growth phenotype prepared as seed candidates for vaccine production. PLoS ONE.

[b16] O’Neill E, Donis RO (2009). Generation and characterization of candidate vaccine viruses for prepandemic influenza vaccines. Curr Top Microbiol Immunol.

[b17] Gubareva LV, Wood JM, Meyer WJ (1994). Codominant mixtures of viruses in reference strains of influenza virus due to host cell variation. Virology.

[b18] Gambaryan AS, Matrosovich TY, Philipp J (2012). Receptor-binding profiles of H7 subtype influenza viruses in different host species. J Virol.

[b19] Suzuki Y, Kato H, Naeve CW, Webster RG (1989). Single-amino-acid substitution in an antigenic site of influenza virus hemagglutinin can alter the specificity of binding to cell membrane-associated gangliosides. J Virol.

[b20] EMA (2013). http://www.ema.europa.eu/docs/en_GB/document_library/Scientific_guideline/2013/03/WC500139747.pdf.

[b21] WHO (2013). http://www.who.int/influenza/vaccines/virus/201302_h5h7h9_vaccinevirusupdate.pdf.

[b22] Jadhao SJ, Achenbach J, Swayne DE, Donis R, Cox N, Matsuoka Y (2008). Development of Eurasian H7N7/PR8 high growth reassortant virus for clinical evaluation as an inactivated pandemic influenza vaccine. Vaccine.

[b23] Ulmer JB, Valley U, Rappuoli R (2006). Vaccine manufacturing: challenges and solutions. Nat Biotechnol.

[b24] Brown LE, Murray JM, White DO, Jackson DC (1990). An analysis of the properties of monoclonal antibodies directed to epitopes on influenza virus hemagglutinin. Arch Virol.

[b25] Takahashi T, Hashimoto A, Maruyama M (2009). Identification of amino acid residues of influenza A virus H3 HA contributing to the recognition of molecular species of sialic acid. FEBS Lett.

[b26] Gambaryan AS, Matrosovich MN, Bender CA, Kilbourne ED (1998). Differences in the biological phenotype of low-yielding (L) and high-yielding (H) variants of swine influenza virus A/NJ/11/76 are associated with their different receptor-binding activity. Virology.

[b27] Guarnaccia T, Carolan LA, Maurer-Stroh S (2013). Antigenic Drift of the Pandemic 2009 A(H1N1) Influenza Virus in a Ferret Model. PLoS Pathog.

[b28] Xu R, de Vries RP, Zhu X (2013). Preferential recognition of avian-like receptors in human influenza A H7N9 viruses. Science.

[b29] Yang H, Carney PJ, Chang JC, Villanueva JM, Stevens J (2013). Structural analysis of the hemagglutinin from the recent 2013 H7N9 influenza virus. J Virol.

[b30] Hensley SE, Das SR, Bailey AL (2009). Hemagglutinin receptor binding avidity drives influenza A virus antigenic drift. Science.

[b31] Chen Z, Baz M, Lu J (2014). Development of a high yield live attenuated H7N9 influenza vaccine that provides protection against homologous and heterologous H7 wild-type viruses in ferrets. J Virol.

[b32] Ramanunninair M, Le J, Onodera S (2013). Molecular signature of high yield (growth) influenza a virus reassortants prepared as candidate vaccine seeds. PLoS ONE.

[b33] Mitnaul LJ, Matrosovich MN, Castrucci MR (2000). Balanced hemagglutinin and neuraminidase activities are critical for efficient replication of influenza A virus. J Virol.

[b34] Myers JL, Wetzel KS, Linderman SL, Li Y, Sullivan CB, Hensley SE (2013). Compensatory hemagglutinin mutations alter antigenic properties of influenza viruses. J Virol.

[b35] Wagner R, Wolff T, Herwig A, Pleschka S, Klenk HD (2000). Interdependence of hemagglutinin glycosylation and neuraminidase as regulators of influenza virus growth: a study by reverse genetics. J Virol.

